# Whole-Genome Sequence of Lactiplantibacillus plantarum Mut-3, Isolated from Indonesian Fermented Soybean (Tempeh)

**DOI:** 10.1128/mra.00513-22

**Published:** 2023-02-22

**Authors:** Ida Bagus Agung Yogeswara, Mariyatun, Putrika Citta Pramesi, Endang Sutriswati Rahayu

**Affiliations:** a Nutrition Department, Universitas Dhyana Pura, Bali, Indonesia; b Faculty of Agricultural Technology, Universitas Gadjah Mada, Yogyakarta, Indonesia; c Center for Food and Nutrition Studies, Universitas Gadjah Mada, Yogyakarta, Indonesia; Indiana University, Bloomington

## Abstract

Lactiplantibacillus plantarum Mut-3 was isolated from tempeh. After whole-genome sequencing, analysis of its possibility as a probiotic candidate was performed using subsystem analysis with RAST with the SEED viewer.

## ANNOUNCEMENT

Lactiplantibacillus plantarum is widely found in fermented foods and in the mammalian gastrointestinal tract (1). In addition, this species commonly colonizes human mucosae of the mouth, vagina, and intestinal tract (2, 3). L. plantarum has been used as a starter culture and has a long history of safe use in food fermentation. Several L. plantarum strains have been isolated from Indonesian fermented foods, and some of them have abilities as probiotic agents and/or producers of γ-aminobutyric acid (GABA) (4, 5). In addition, some strains isolated from other fermented foods were reported to produce bacteriocins (6).

L. plantarum Mut-3 was isolated from tempeh. The tempeh was bought in a traditional market in Yogyakarta, Indonesia. Isolation started with the incubation of tempeh with MRS agar at 30°C for 3 days. After colonies appeared, pure cultures were isolated and stored as frozen stocks (−80°C) in MRS broth with 10% added glycerol. The strain was deposited at the Center for Food and Nutrition Studies, Universitas Gadjah Mada (Yogyakarta, Indonesia), as FNCC 0246. Before isolation, L. plantarum Mut-3 was prepared by incubating the strain in MRS broth (Merck, Germany) at 37°C for 24 h. The isolate was previously identified as Lactiplantibacillus plantarum subsp. *plantarum* Mut-3 using 16S rRNA gene sequencing (7). This study aimed to report the whole-genome sequence of L. plantarum Mut-3 and investigated its ability as a probiotic strain. The results of this research will be of great importance to academic institutions and should facilitate the use of the strain in clinical trials. In addition, food industries that produce probiotic foods might use the results of this study when considering probiotic strains for their products.

Before the DNA extraction, the culture strain was cultivated after growth of the strain in MRS broth (catalog number 110661; Merck, Darmstadt, Germany) and incubated at 37°C for 24 h. The genomic DNA was extracted using the SDS method (8), and the harvested DNA was detected using agarose gel electrophoresis and quantified with a Qubit 2.0 fluorometer (Thermo Fisher Scientific, USA). For library construction, 1 μg DNA per sample was used as the input material for the DNA sample preparation. The sequence libraries were generated using the NEBNext Ultra DNA library preparation kit for Illumina (New England Biolabs, USA) according to the manual and index codes. The DNA sample was fragmented to 350 bp by sonication for Illumina sequencing with further PCR amplification. The products were purified, and libraries were analyzed for size distribution with an Agilent 2100 Bioanalyzer and quantified using real-time PCR. The whole-genome sequencing was performed using an Illumina NovaSeq system (paired-end 150-bp reads). The circularization of the genome was done computationally.

The raw data obtained by sequencing had a certain proportion of low-quality data. To ensure the accuracy and reliability of the results of subsequent analyses, the original data went through sequence quality control. First, reads with low-quality nucleotides (mass values of ≤20) as >40% of the reads were removed. Then, reads containing >10 bp of N nucleotides were removed. Reads that overlapped with the adapter for >15 bp were also removed. When the sample was contaminated by the host (i.e., minigenome), the sample was subjected to a BLAST search with the host data to filter out reads that might have originated from the host.

Assembly of the clean data used three different software packages, i.e., SOAPdenovo (version 2.04) (9, 10), SPAdes (11), and ABySS (12). Subsequently, the results from the different software processes were integrated using CISA software (13). All of the software programs used in this study used default parameters except where otherwise noted.

The resulting genome components, which mostly consist of functional regions, were annotated using repeat annotation statistics, noncoding RNA (ncRNA) annotation statistics, and coding gene annotation statistics. All of the protein sequences were aligned to the genome sequences using BLAST, and then GeneWise was used for gene structure prediction. The Gene Ontology (GO) (14), KEGG (15, 16), Clusters of Orthologous Genes (COG) (17), NCBI nonredundant (18), Pfam (19), and Swiss-Prot (20) databases were used for general gene annotation. Subsystem analysis was performed with Rapid Annotation using Subsystem Technology (RAST). According to the RAST quality report (https://www.theseed.org/wiki/RAST_Quality_Report), RAST can recognize six classes of overlaps, namely, embedded protein-encoding genes (PEGs), bad RNA overlaps, convergent overlaps, divergent overlaps, same-strand overlaps, and impossible overlaps.

The genome of strain L. plantarum Mut-3 was 3,249,982 bp in total length, in a circular chromosome with a GC content of 44.4%. During the assembly, the number of contigs with PEGs obtained was 29, and the sequencing depth was 351×. In total, 3,073 coding sequences (CDSs) and 76 RNA genes were predicted. Strain L. plantarum Mut-3 carries three bile salt hydrolase genes and a glutamate decarboxylase gene (*gadB*) with a length of 1,410 bp, encoding 470 amino acids; *gadB* genes are responsible for the acid resistance system through the glutamate-dependent acid resistance (GDAR) system and GABA production (21). The *gadB* gene of L. plantarum Mut-3 is located between phosphoenolpyruvate carboxykinase and a hypothetical protein gene.

The subsystem analysis using RAST with the SEED viewer revealed that strain L. plantarum Mut-3 contains 1,397 subsystem features. The L. plantarum Mut-3 genome includes 56 hypothetical genes and 1,217 nonhypothetical genes. Genes that are related to probiotic properties were identified as being present in the genome of L. plantarum Mut-3. In this regard, exopolysaccharide-encoding genes may be involved in biofilm formation and may offer protection from phage infection and heavy metals (22). We also annotated genes encoding proteins predicted to be involved in bacteriocin (lactococcin, cloacin, and linocin) production (23). The annotation found four osmoregulatory system (ABC transporter) *opuC* genes (*opuCA*, *opuCB*, *opuCC*, and *opuCD*), which may contribute to the survival ability of the strain under the harsh conditions of the gastrointestinal tract. A previous study investigating the osmoprotectant management of Bacillus subtilis reported that OpuC can import the osmoprotectants choline and glycine betaine (24). Furthermore, the gene encoding dTDP-glucose-4,6-dehydratase was detected in the genome of L. plantarum Mut-3 and may be responsible for the acid tolerance of the strain.

### Data availability.

The complete genome sequence of L. plantarum Mut-3 has been deposited in GenBank under accession number JAMDWZ000000000. The raw data can be accessed under SRA accession number SRR19627660. The BioProject and BioSample accession numbers are PRJNA832026 and SAMN27771036, respectively.
